# Perspectives of deprived patients on diabetes self-management programmes delivered by the local primary care team: a qualitative study on facilitators and barriers for participation, in France

**DOI:** 10.1186/s12913-020-05715-3

**Published:** 2020-09-11

**Authors:** Emmanuel Allory, Hélène Lucas, Arnaud Maury, Ronan Garlantezec, Candan Kendir, Anthony Chapron, Laure Fiquet

**Affiliations:** 1 Department of general practice, University of Rennes 1, F-35000 Rennes, France; 2 Univ Rennes, CHU Rennes, Inserm, CIC 1414 (Centre d’Investigation Clinique), F-35000 Rennes, France; 3 Department of General Practice, Medicine Faculty of Rennes, 2, Avenue of Pr Léon Bernard, 35043 RENNES Cedex, Rennes, France; 4 CHU de Rennes, Univ Rennes, Inserm, EHESP, Irset (Institut de recherche en santé, environnement et travail) - UMR_S 1085, F-35000 Rennes, France; 5 École des hautes études en santé publique (EHESP), Saint-Denis, France

**Keywords:** Self-management, Patient education as topic, Vulnerable population, Diabetes mellitus, Primary health care

## Abstract

**Background:**

Diabetes self-management education (DSME) is an effective intervention for patients with type 2 diabetes mellitus (T2DM); nevertheless, patient participation in this type of programme is low. Implementation of DSME programmes in primary care practices by the local multi-professional team is a potential strategy to improve access to DSME for T2DM patients. The aim of this study was to identify perceived facilitators and barriers by patients to participation in local DSME delivered by primary care professionals in France.

**Method:**

T2DM patients, informed and recruited during consulting with their usual care provider, who had attended a structured and validated DSME programme delivered by 13 primary care providers within a multi-professional primary care practice in a deprived area of 20,000 inhabitants, were invited to participate in this study. A qualitative study with semi-structured, in-depth interviews was conducted with study participants, between July 2017 and February 2018. A reflexive thematic analysis of the interviews was carried out. Coding schemes were developed to generate thematic trends in patient descriptions of facilitators and barriers to DSME participation.

**Results:**

Nineteen interviews (mean length 31 min; [20–44 min]) were completed with T2DM patients. Four themes on facilitators for programme participation emerged from the data: geographical proximity of a DSME programme held in the local multi-professional primary care practice; effective promotion of the DSME programme by the local multi-professional team; pre-existing relationship between patients and their healthcare providers; and potential to establish new social interactions within the neighbourhood by participating in the programme. Three themes on barriers to attendance emerged: integrating the DSME programme into their own schedules; difficulties in expressing themselves in front of a group; and keeping the motivation for self-managing their T2DM.

**Conclusions:**

From the patient perspective, the programme geographical proximity and the pre-existing patient-healthcare provider relationship were important factors that contributed to participation. Healthcare providers should consider these factors to improve access to DSME programmes and diabetes self-management in deprived populations. Longitudinal studies should be performed to measure the impact of these programmes.

## Background

The Global Burden of Disease study showed that type 2 diabetes mellitus (T2DM) burden has increased by more than three times between 1980 and 2014 [1]. TD2M prevalence and complications are higher in vulnerable populations (e.g. individuals who are socioeconomically deprived and without healthcare insurance; racial and ethnic minorities) than in the general population [2–4]. To reduce this burden, diabetes self-management education (DSME) has been proved effective and is recommended by international guidelines [5]. However, literature data have shown a low referral rates to DSME and low attendance by the referred individuals [6–8]. Therefore, many studies focused on the facilitators and barriers to DSME attendance [8–14]. They found that attendees are more interested in participating when the DSME programme addresses an unmet need, for instance information less centred on medications and more on lifestyle, and schematic presentation of the many things one should know [10]. Moreover, healthcare providers are considered as great information providers and supporters for DSME programme attendance. As mentioned by Saha et al. in their systematic review about the state of art of DSME programes in European Union, the attendance at the programme could be improved by increasing accessibility of the population to these programmes, as well as by tailoring the programmes to specific patient groups [15]. Indeed, delivery by educators who are integrated in the attenders’ primary care team is considered a facilitator [12]. Finally, one study conducted among healthcare providers in a deprived neighbourhood in Sweden showed that cultural knowledge of the educators about the population helps them to better fit the DSME programme to the needs of individuals [13]. On the other side, many barriers have been highlighted mostly related to difficulties in accessing such programmes [8, 10]. To overcome these difficulties, some authors recommended to better embed and integrate DSME in the T2DM routine care pathway [8–10].

Three recent studies conducted in the United-States and Canada found that the development of DSME programmes in primary care practices is considered one of the key solutions to increase their attendance rate [12, 14, 16]. A systematic review from 2001, examining the effectiveness of the intervention that aims to improve the management of diabetes amongst primary care providers, showed that primary care centres offer patient-centred care with a holistic approach, and have a significant role in chronic disease management [17]. They are easily accessible by the population and favour the access to healthcare better than hospitals [18]. Some studies showed that the probability to receive good quality care in primary care practices was higher in centres with a DSME programme [19]. However, an international review of articles in five languages found that only 20.3% of DSME programmes are held in primary care practices [20]. This results in lack of referral to the programmes or incomplete follow-up of attendance [21].

Since 2007 and in line with the international recommendations [22], the French Health Authority (Haute Autorité de Santé; HAS) considers DSME programme accessibility a priority. These programmes, structured according to the HAS guidelines, need to be authorised and are evaluated every 4 years [23]. They can be performed in different settings, including by telephone and online (e.g., the “Sophia” programme developed by the health insurance fund), at hospitals or in diabetes education centres, which are located in the main city of each French region, and in primary care practices. All these DSME programmes are free of charge. A recent audit showed that in France, less than 7% of DSME programmes are held in primary care settings [24]. Moreover, only 1% are organised by multi-professional primary care practices, although multi-professional collaboration is becoming more common among local primary care professionals, resulting in more collaborative activities, such as DSME programmes [24].

Studies exploring the perspectives of individuals attending DSME programmes in their own multi-professional primary care practice are scarce. Assessing and understanding the facilitators and barriers to DSME attendance in their own multi-professional primary care practice would improve the T2DM care pathway. This study investigated how the development and implementation of a DSME programme provided by the local multi-professional primary care practice can influence DSME attendance from the point of view of patients with T2DM. The objective of this study was to analyse the perspectives of deprived people with T2DM concerning facilitators and barriers to attend a DSME programme at their own multi-professional primary care practice.

## Methods

### Design

A qualitative and exploratory study was carried out between July 2017 and February 2018. A reflexive thematic analysis was chosen as a theoretical approach. The flexibility of this theory allowed us to identify themes within and across data in relation to the participant’s experience, perspectives, and practices [25]. The Consolidated Criteria for Reporting Qualitative research (COREQ) checklist was used to provide a summary of our manuscript [26] and is shown in Additional file 1. This study was approved by the Rennes University Hospital ethics committee on September 2, 2017 (Number 17.47).

### Setting and DSME programme

Our multi-professional primary care practice, (Maison de Santé Pluriprofessionnelle; MSP), is the primary care setting in the socioeconomically deprived neighbourhood of Villejean (Rennes, France) that is characterized by a high index of poverty, high unemployment rate, low doctor’s consultation rate, and high foregoing medical care [27]. The practice includes 73 primary healthcare professionals from 16 different professions, and targets a population of 20,000 inhabitants. This multi-professional primary care practice has developed an innovative and adapted DSME programme based on the HAS guidelines. This DSME programme was validated by the Local Health Authority (Agence Regionale de Santé; ARS) and involves local primary care providers. This programme, which started in 2017, includes eight weekly workshops of 90 min and is conducted twice per year. The topics of the eight workshops on diabetes are: disease representation, different pharmacological treatments, self-management of foot care, balanced diet, importance of carbohydrates in the diet, physical activity, eye care, and supplies and equipment for diabetes self-management (Additional file 2).

People with diabetes listed in our primary care practice received information about the DSME programme from their healthcare provider. Healthcare providers recruited participants during their usual consultations (e.g. follow-up consultation with the family doctor or consultation at the pharmacy). Interested people had an individual conversation with a team member before the DSME programme start to fix personal objectives that were used to prepare a personalised attendance programme to the different workshops. When people met all their personal objectives, they had a new conversation with the same team member to define the completion of the DSME programme. The workshop contents were created by 13 healthcare providers from the local primary care team who worked collaboratively on different parts of the programme. They all contributed to patient recruitment for the DSME programme and delivered some workshop sessions. Ten of these professionals met the patients individually before the programme start (to define their personalised objectives) and at the end of the programme. Approximately 30 patients attend the programme each year. Thirteen healthcare providers received a multi-professional DSME training by the French association for the development of structured education (AFDET).

### Participants and methods

Semi-structured individual in-depth interviews were carried out with patients who attended the programme in 2017. It started in 2017 and was delivered twice during the year: March–April 2017 and October–November 2017. After completing the DSME programme, all participants were contacted by phone (HL) (two attempts for each participant) and an appointment time was planned for a face to face interview, in the case of positive answer. To increase the participants’ recruitment and to make them feel comfortable, they could choose the interview place, either at home or at the primary care practice.

### Data collection

After an initial training with a senior lecturer (LF) who is expert in qualitative research, one of the authors (HL) conducted the interviews. She introduced herself as a healthcare student. All interviews were digitally recorded and transcribed verbatim. Field notes were made throughout. Written informed consent was obtained from each interviewee before the interview for all the information used for the study purposes.

The interview guide was developed based on the literature review performed at the beginning of the study (EA, HL), and after discussion with a DSME expert (AC) and all co-authors (AM, CK, LF). The final version was pilot tested with patients with T2DM from another primary care practice. The interview guide included three sections: 1) description of their type 2 diabetes history (how it started, how it has changed over time); 2) reasons for participating in the DSME programme, and the obtained benefits; and 3) factors that motivated their attendance. The first section was used as an icebreaker between interviewer and interviewee.

At the end of each interview, the interviewer asked questions to the interviewee to answer some additional questions about the demographic data (age, sex, occupation, education level, and country of origin). She also extracted the interviewee’s HbA1c value at the programme start and the year of T2DM diagnosis from the medical record (Table 1). The interviewees’ participation to other DSME programmes (provided by the diabetes education centre or health insurance company) was noted. Finally, the participants’ deprivation level was calculated with the Evaluation of Deprivation and Inequalities in Health Examination Centres score (Evaluation de la Précarité et des Inégalités de santé dans les Centres d’Examens de Santé; EPICES) [28]. The EPICES score is an individually applied standardised score developed by a panel of French National Health Insurance experts in 2002. It includes 11 items about isolation, health insurance status, economic status, social support, and leisure activities. The total score varies between 0 and 100, with a cut-off of 30.17. Participants with a score above this threshold are considered deprived.

**Table 1:** Participants’ characteristics (*n* = 19)

Participant code	Sex	Age	EPICES score	Interview duration (min)	Previous DSME	Education level	Country of origin	HbA1c (at the DSME programme start) (mmol/mol – %)	Year of T2DM diagnosis
P1	M	79	7.1	44	D35	Low	France	57–7.4	1987
P2	M	70	55.03	31	No	Low	Algeria	50–6.7	1996
P3	M	62	71.6	42	D35	Low	France	55–7.2	1985
P4	W	57	65.09	32	No	High	Burundi	60–7.6	2012
P5	M	58	65.09	29	No	Middle	France	52–6.9	2012
P6	W	61	56.8	22	No	Middle	France	49–6.6	2016
P7	W	61	38.46	27	D35/HEC	Middle	France	64–8	2008
P8	W	75	52.07	34	D35	Middle	Serbia	61–7.7	2006
P9	M	67	22.48	43	HEC/Sophia	Middle	France	60–7.6	1992
P10	M	70	38.46	25	No	High	DRC	46–6.4	2016
P11	W	55	39.05	26	No	Low	France	49–6.6	2014
P12	W	48	25.44	23	No	Low	Haiti	42–6	2015
P13	M	63	56.22	39	No	Middle	Laos	60–7.6	2015
P14	W	47	100	20	D35/HEC	Low	DRC	81–9.6	2012
P15	M	54	29	39	HEC	Middle	Morocco	50–6.7	2017
P16	W	55	55.62	37	No	Middle	DRC	65–8.1	1985
P17	W	41	53.85	38	No	Middle	France	109–12.1	2000
P18	M	55	30.18	24	Sophia	Middle	Suriname	54–7.1	1988
P19	W	65	80.47	21	No	Middle	France	66–8.2	1998

*P* Participant, *W* Woman, *M* Man, *DSME* Diabetes Self-Management Education, *TD2M* Type 2 Diabetes Mellitus, *D35* (Diabetes 35) = collective DSME sessions by a diabetes education centre in another centre; HEC (Health Examination Centre) = collective DSME sessions organised by a health insurance company in another centre; Sophia: individual DSME sessions by phone organised by a health insurance company; DRC: Democratic Republic of Congo. Education level, low: primary school, middle: secondary and high school, high: higher than high school; HbA1c: usual objective for people with type 2 diabetes is 53 - 7 (mmol/mol - %)

### Data analysis

All recorded interviews and field notes were transcribed verbatim using the Microsoft Office Word software (HL) and were not returned to participants for comments or corrections. The thematic analysis was conducted concomitantly with the interviews to answer the research question. Two researchers (EA, HL) independently coded these interviews in an active and reflexive process as defined by Braun and Clarke [25]. After familiarising with the interview content, each researcher independently generated the initial codes of the interview. Each researcher compared the analysis of the last interview with that of the previous interviews in an iterative approach. These codes were then discussed by the two researchers, interpreted, and grouped in themes. In the case of conflict, a third researcher (LF) independently analysed the interview, and contributed to the discussion. Data saturation, defined as the absence of new ideas emerging from the analysis, was achieved after the seventeenth interview (no new code from the last two interviews). The report was then produced [29, 30].

## Results

In total, 27 individuals attended the DSME programme in 2017. All of them were literate in French, and 19 could be interviewed (70.4%). The mean interview length was 31 min [20–44 min]. The reasons for non-participation were being abroad (*n* = 2), refusal to be recorded (*n* = 1), registered but finally did not attend the DSME programme (*n* = 2), and could not be reached (*n* = 3). The 19 interviewed participants (nine men and ten women; mean age: 60.1 ± 9.7 years) came from nine different countries (France, Algeria, Burundi, Serbia, Democratic Republic of Congo, Haiti, Laos, Morocco, Suriname), and 58% (*n* = 11) had never attended a DSME programme before. Their mean ± standard deviation EPICES score was 50 ± 22.3 points, and 78.9% (*n* = 15) of participants were deprived according to this score (Table 1).

On the basis of the interviews’ analysis, we generated two main themes (facilitators and obstacles to attendance), each containing subthemes, presented in Table 2. In the following section, we describe the themes with quotes from interviewees.

**Table 2:** Themes and subthemes generated by analysis of the interviews

Themes	Subthemes
Facilitators	Geographical proximity of the primary care practice was a facilitating factor
	The DSME programme promotion by the multi-professional team to the participants was effective
	The existing relationship with the primary care providers was a motivating factor
	Social relationships with people from the same neighbourhood were a key point of local DSME programmes
Barriers	Integrating the DSME programme in their own schedule was difficult
	Self-expression was a barrier for some participants
	Keeping their motivation to manage T2DM was difficult

*DSME* Diabetes Self-Management Education, *T2DM* Type 2 Diabetes Mellitus

### Facilitators to participation

#### The geographical proximity of the primary care practice is a facilitating factor

Patients, especially those who never went to a DSME programme before, found that geographical proximity was an important point because it facilitated the access and attendance to the DSME sessions. Concomitantly, they criticized the difficulty of access to programmes held far away from their neighbourhood.

‘First I was not far! I am living just nearby! So, first, this point was important [ …] It is true that it was easier to go to a close-by place (P6)’.

They explained that the constraints imposed by their daily life activities hampered attendance when the sessions were at a distant centre. Moreover, they found sensible and beneficial to receive both preventive and curative care in the same place.

‘What is great in this primary care practice is that everything is here; it is the greatest aspect. We have everything in the same place. We do not have to run to different places. This is important. I have a driving licence, but no car. So, it is great to know it is not far (P6)’.

Another advantage to have DSME sessions at their own primary care practice was that this was a familiar place to all of them, thus avoiding the fear of an unknown place.

‘In the programme, there were three nurses whom I know well, including ‘X’, who is great and nice. It is almost like a family (P4).’

#### Promotion of the local DSME programme by the multi-professional team to the target population is effective

Patients who previously went to DSME sessions outside the primary care practice mentioned the easier access to information about the local DSME programme through their own primary care professionals, compared with the other programmes.

‘I have never received this kind of information about a programme. It was the first time (P3)’.

The information about the DSME programme was well disseminated by the multi-professional team, allowing a large diffusion and an effective recruitment.

‘My family doctor said: “There is a woman just at the end of the corridor; she is a nurse; we go and talk to her.” The nurse took care of me, and suggested to me to go to this patient education (P10)’.

Moreover, the healthcare providers remembered that their patients went to the local DSME programme and discussed with them about it during their usual consultations.

‘My family doctor often asks me whether I am still going to the sessions. For now, yes I continue to follow the programme (P11)’.

#### The existing relationship with the primary care providers is a motivating factor

Patients said that the idea of a local DSME programme with known and motivated professionals as diabetes educators was a motivating factor, and they appreciated their involvement and the ‘family atmosphere’.

‘When you know someone, it is easier. I have known this pharmacist for 20 years […], when she told me “I will be there”, it was another reason for me to go (laugh) (P15)’.

The multi-professional approach played a role in the patient recruitment and in improving their knowledge about the local healthcare professional network.

‘It is a great programme. We have the nurses, the chiropodist…. It concerns many health professions involved in diabetes (P9)’.

Patients appreciated the healthcare providers’ knowledge about their socioeconomic and cultural environment and their patient-centred approach that allowed developing a more trustful relationship.

‘I know well Dr X, he is a nice guy who knows the neighbourhood well, who knows our problems. He is the first person to talk about the social difficulties in our area (P1)’.

#### Social relations with people from the same neighbourhood are a key point of local DSME programmes

Although it was difficult for some patients to interact in a group, they found beneficial to meet other people with T2DM who lived in the same area. The DSME programme reduced their social isolation and created new social relationships.

‘It was a pleasure for me to meet other patients from the neighbourhood who were talking about diabetes, and also to make new friends. Friends by whom I feel I am understood and whom I can understand (P12)’.

Attendees appreciated the possibility to exchange with people who had the same culture and lived in the same environment.

‘I knew nearly three-quarters of the other patients; that was a good starting point to create a great atmosphere (P3)’.

Recognising their own habits and difficulties in other participants helped them to accept their own disease.

‘It is also interesting to see friends who also have diabetes with different habits. Everyone has his/her own path (P19)’.

### Barriers to participation

#### Integrating the DSME programme in their schedule is difficult

Participants found difficult to integrate the programme in their schedule for professional or family reasons.

‘But after the start of the programme, I got a job, my first job, I couldn’t refuse it! Therefore, I could not attend all the programme sessions (P7)’.

The programme timetable created difficulties for some participants whose agenda was not flexible.

‘Because I am working only in the afternoon. And all the sessions were in the afternoon. So, I couldn’t go to many of them (P18)’.

Moreover, participants often had many medical visits and therefore they did not consider the DSME programme a priority.

‘I am busy these days, I have tooth problems. I have an appointment every week. So, I made this dental visit a priority (P13)’.

#### Self-expression is a barrier for some participants

For some participants, talking about their own disease in front of a group could represent the main obstacle. During the sessions, some participants came up with ideas about practical activities. For instance, instead of explaining orally their difficulties with glucometers, they asked to be observed while measuring their glucose level and to be directly helped to correctly use the device.

‘It is not easy for people to come and participate in the programme. They are afraid to meet each other. They are afraid to talk about themselves, about their disease, about their intimate life! It is not easy! (P4)’.

‘I don’t want to go because there is a lot of talking to do. If I don’t talk with the others, if they don’t like my behaviour, they will not be happy and they will get offended! That’s why I don’t want to go! (P13)’

Knowing the healthcare providers was an advantage, but did not completely stop the fear of the first move by participants.

‘Definitely, it is not always easy to come to the first meeting; you have to take the first step. You have to motivate yourself, and this is the hardest part. But when I was there, I was feeling good (P19)’.

#### Keeping the motivation to manage T2DM is difficult

Some participants expressed some weariness concerning the T2DM management. The programme was yet another “task” to do and was difficult to integrate in their T2DM management.

‘I know there was a time I was tired about diabetes! I could be told about it but I didn’t care. But now, I got to a certain age and I want to take care of myself (P17)’.

Moreover the programme benefits were not always fully clear to the participants either because of wrong perception of the programme content, or because they did not see any advantage for them in attending.

‘As long as I feel physically fine, I don't really feel the need to go, sit down, listen, "be careful", the red light isn't on yet to push me to go there! (P18)’.

‘Like I said, I was not really into it. I did not feel like I needed it. I felt like I was healthy (P19)’.

## Discussion

Our study on the perspectives of deprived individuals who attended a T2DM programme in their own multi-professional primary care practice revealed the existence of facilitators and also barriers to their participation in such programme. Among facilitators, four themes were identified: i) geographical proximity (knowing the area and limited need of transportation were features that facilitated the attendance); ii) easier access to the information about the DSME programme and higher effectiveness of its promotion through the multi-professional primary care providers; iii) existing trustful relationship with the healthcare providers and their holistic approach; and iv) possibility to improve their social interactions by creating or strengthening the link with other patients when attending a DSME programme in their own neighbourhood. The three main barrier themes were: i) difficulties to integrate the DSME programme in their schedule, despite the geographical proximity; ii) difficulties in self-expression (knowing the health educators did not decrease the fear of the first move); and iii) difficulties in keeping the motivation for diabetes self-management.

### Comparison with existing literature

This study described the facilitators and barriers perceived by deprived people for attending a local DSME programme developed in their own primary care practice. In the literature, barriers to DSME programmes are described using the terms ‘can’t go’ (people who choose not to attend for logistical reasons) and ‘won’t go’ (people who do not attend for emotional or cultural reasons) [8, 10]. In our study, access was facilitated by holding the workshops in the local primary care centre (i.e. geographical proximity) and by the participants’ familiarity with the place and people providing and attending the DSME programme (regular healthcare providers, friends from the neighbourhood).

Previous studies also highlighted the low referral rate to DSME programmes [6, 21] and the lack of communication about the programme [10]. Our interviewees said that access to information on their local DSME programme was easier compared with other programmes they attended previously [11]. Effective promotion is important to improve the knowledge on these programmes by healthcare professionals and targeted populations [10] and consequently to increase the referral rate to such programmes by healthcare providers [21]. Our study suggests that the involvement of local healthcare providers in the programme increases their engagement in promoting it. Therefore, the direct involvement of multi-professional primary care practices appears to be a strategy to improve DSME attendance in deprived areas.

As vulnerable people are particularly affected by diabetes and comorbidities, access to health education is an important priority [2, 3, 31]. However, many barriers exist especially among healthcare providers who feel inadequately equipped to deal with the cultural differences [13]. Our interviewees found that knowledge about their socio-economic and cultural environment by the educators was a facilitator. This knowledge seems to improve the mutual understanding between vulnerable patients and healthcare providers [32], and could contribute to reducing the cultural barriers between them [13]. Access to the healthcare system and mobility are often limited for vulnerable populations [2, 31, 33]. Our study shows that easier geographical access can help deprived populations to attend a DSME programme. Moreover, holding such programmes in a local centre can help vulnerable people to socialize, and to prevent/limit social isolation [34].

### Implications

Given the importance of developing patient education in the framework of chronic diseases, our findings suggest some practical considerations for future health education programmes. First, as mobility limitations affect the health of city dwellers [33], DSME programmes in primary care centres should be encouraged and supported to increase the participation rate. Primary care, defined by its spatial accessibility, has a special place for improving the access to DSME, particularly by patients who live in deprived areas [18, 35]. However, a systematic review showed that due to organizational deficiencies, primary care teams may experience difficulties in self-managing their own health education programmes [36]. Indeed, such programmes can be maintained only if there is a central support to the primary care team [37]. Therefore, only a strong support from policy makers can help primary care providers to initiate and maintain DSME programmes.

Second, as the existing social difficulties and concerns can limit social access and promote isolation of deprived populations, DSME programmes should consider the development of new social links as an objective for participants [38]. This objective, which goes beyond the medical aspect, might also help patients to attend the DSME programme and to strengthen disease self-management through peer support [34, 39]. Longitudinal studies and more comprehensive evaluations of DSME programmes in primary care are now needed to better identify their impact on deprived patients.

### Limitations

The study has some limitations. First, it focused on participants who attended the same DSME programme in a single primary care practice. Therefore, our results cannot be generalised. However, our study design focused on examining a unique and specific population which is usually difficult to reach out and improving attendance to self-management programme for this population. Additionally, our study setting was a local primary care practice where there is few information in the literature. In their studies, Albano et al. and St Pierre et al. mention the scarcity of DSME programme development and evaluation in primary care practice [20, 24]. Thus, we believe our study amongst deprived population in a primary care practice brings an added value to the literature. Second, the findings represent the opinions of the patients who participated in the programme. Their barriers were integrating the DSME programme in their own agenda, self-expression and keeping the motivation in the long-term. We did not collect any data among non-attendees. Therefore, we do not know anything about the reasons of non-attendance and of refusing the interview by some people who attended the programme. However, in a systematic review in 2017, Horigan et al. showed that the difficulties listed by people who did not attend a DSME programme were similar to those experienced by people who attended and presented these barriers in two categories: ‘could not attend’ and ‘would not attend’ [8]. Nevertheless, a study which will focus on non-attendees could bring valuable information that could be used to improve attendance.

## Conclusion

Our study analysed the DSME attendance facilitators and barriers reported by patients who participated in a DSME programme developed by and implemented at their multi-professional primary care practice. The positive role of geographical proximity should encourage more primary care providers to develop DSME programmes. Moreover, the local multi-professional team efficiently disseminated information about the programme.

The facilitating factors and barriers that emerged from our study should be taken into account for developing new DSME programmes to target deprived populations especially in primary care contexts. To our knowledge, the literature still lacks information about the long-term impacts of DSME programmes in primary care. Therefore, we highly encourage assessment of these programmes in primary care in longitudinal studies.

## Supplementary information

**Additional file 1.** Consolidated criteria for reporting qualitative research (COREQ): 32-item checklist.

**Additional file 2.** Details of the diabetes self-management programme at the primary care practice.

## Data Availability

The datasets used and analysed during the current study are available from the corresponding author on reasonable request.

## Abbreviations

AFDET: Association Française pour le Développement de l’Education Thérapeutique (French association for the development of structured education)
COREQ: Consolidated Criteria for Reporting Qualitative Research
DSME: Diabetes Self-Management Education
EPICES: Evaluation de la Précarité et des Inégalités de santé dans les Centres d’Examens de Santé (Evaluation of Deprivation and Inequalities in Health Examination Centres)
HAS: Haute Autorité de Santé (French Health Authority)
MSP: Maison de Santé Pluriprofessionnelle (multi-professional primary care practice)
T2DM: Type two Diabetes Mellitus
