# Supplementary material for: Perspectives of deprived patients on diabetes self-management programmes delivered by the local primary care team: a qualitative study on facilitators and barriers for participation, in France
Source: BMC Health Serv Res. 2020 Sep 11;20:855. doi: 10.1186/s12913-020-05715-3 (PMC7488295; doi:10.1186/s12913-020-05715-3)
Supplement: Supplementary file 1 — Additional file 1. Consolidated criteria for reporting qualitative research (COREQ): 32-item checklist. [file 12913_2020_5715_MOESM1_ESM.docx]

| **Additional file 1.** Consolidated criteria for reporting qualitative research (COREQ): 32-item checklist | |
| --- | --- |
| **Item** | **Guide questions/description** |
| **Domain 1: Research team and reflexivity** | |
| *Personal characteristics* | |
| 1. Interviewer/facilitator | Interviews were conducted by one of the authors ( H. Lucas) |
| 1. Credentials | H. Lucas, resident doctor in family medicine |
| 1. Occupation | At the time of the study, H. Lucas was a resident in family medicine, University of Rennes 1 |
| 1. Gender | H. Lucas is a woman |
| 1. Experience and training | H. Lucas is a doctor in family medicine. She followed a course by a senior researcher, expert in qualitative research, during her residency |
| *Relationship with participants* | |
| 1. Relationship established | Participants were unknown to the researcher before the research start |
| 1. Participants’ knowledge of the interviewer | Participants were informed about the study prior to the interviews through a phone call by the interviewer. To increase the participants’ recruitment rate and to make them feel comfortable, they could choose the interview place (home or practice). |
| 1. Interviewer’s characteristics | The interviewer introduced herself as a student without specifying her medical background, in order not to be perceived as a family doctor, a status that could make participants uncomfortable |
| **Domain 2: Study design** | |
| *Theoretical framework* | |
| 1. Methodological orientation and theory | Researchers used the reflexive thematic analysis as theoretical background. |
| *Participants’ selection* | |
| 1. Sampling | Participants were recruited from all people who attended the DSME programme |
| 1. Method of approach | Participants were invited by phone, using the contact information they gave. An appointment time was planned for the interview, in the case of positive answer. If the participant was unavailable, a voice message was left in the answering machine, if possible (two attempts for each participant). |
| 1. Sample size | 19 |
| 1. Non-participation | 27 participants were initially approached. The reasons for non-participation were: being abroad (n=2), refusal to be recorded (n=1), registered but finally did not attend the DSME programme (n=2), and could not be reached (n=3). |
| *Setting* | |
| 1. Setting of data collection | To make the participants comfortable, they were interviewed either at home or at the primary care practice, according to their choice |
| 1. Presence of non-participants | Only the interviewer and the participant were present at the interview |
| 1. Description of sample | The study samples were patients with T2DM who went to a structured and validated DSME programme at the primary care practice in 2017. |
| *Data collection* | |
| 1. Interview guide | The interview guide was developed based on a literature review performed at the beginning of the study and after discussion with a DSME expert and all co-authors. The final version was pilot tested with patients from another primary care practice. |
| 1. Repeat interviews | No repeat interview was planned or carried out. |
| 1. Audio/visual recording | All interviews were digitally recorded and transcribed by the interviewer. |
| 1. Field notes | Field notes were made throughout the interviews. |
| 1. Duration | The average interview duration was 31 minutes, with a maximum of 44 and a minimum of 21 minutes. |
| 1. Data saturation | In reflexive thematic analysis, the saturation is not an issue to discuss. |
| 1. Transcripts returned | Transcripts were not returned to the participants |
| Domain 3: Analysis and findings | |
| *Data analysis* | |
| 1. Number of data coders | E. Allory (associated professor at the Family Medicine Department of Rennes University, expert in qualitative research) and the interviewer did the coding. |
| 1. Description of the coding tree | Authors met regularly to discuss interviews, coding and themes. |
| 1. Derivation of themes | Themes were partly identified from the literature and partly derived from the interview data. |
| 1. Software | No software was used. The analysis was done manually. |
| 1. Participant checking | Participants were not invited to check the analysis |
| *Reporting* |  |
| 1. Quotations presented | Quotations from participants were used and assigned individual numeric codes to respect the interviewees’ anonymity (e.g.,: P1, P6…) |
| 1. Data and findings consistent | There was consistency between data and findings. |
| 1. Clarity of major themes | Major themes were generated, presented, and illustrated with quotes |
| 1. Clarity of minor themes | Minor themes were analysed, but not presented due to lack of space |
