# Supplementary material for: Perspectives of deprived patients on diabetes self-management programmes delivered by the local primary care team: a qualitative study on facilitators and barriers for participation, in France
Source: BMC Health Serv Res. 2020 Sep 11;20:855. doi: 10.1186/s12913-020-05715-3 (PMC7488295; doi:10.1186/s12913-020-05715-3)
Supplement: Supplementary file 2 — Additional file 2. Details of the diabetes self-management programme at the primary care practice. [file 12913_2020_5715_MOESM2_ESM.docx]

| **Additional file 2.** Details of the diabetes self-management programme at the primary care practice | | |
| --- | --- | --- |
| Programme providers | Two family physicians  One chiropodist  Two nurses  One pharmacist  One laboratory technician | One dentist  Two physiotherapists  Two expert participants  One medical coordinator |
| Programme modalities | Eight weekly workshops of 90 minutes, leaded by two different healthcare providers of the team. Each participant had an individual conversation with a team member before the beginning and after the programme end, to fix the participation objective(s). | |
| Self-management programme content | 1. Analysis of diabetes representation among participants 2. Pharmacological treatments for diabetes 3. Self-management of foot care 4. Balanced diet 5. Importance of the carbohydrates in the diet 6. Place of physical activity in daily life 7. Eye care for people with diabetes 8. Supplies and equipment, such as needles and injectors, for diabetes treatment | |
| Self-management programme promotion | 1. By the healthcare providers during consultations 2. With posters in the primary care practice | |
